# Comparing Competitive and Recreational Table Tennis Training: Impacts on Quality of life in Older Adults - A Randomized Controlled Trial

**DOI:** 10.12688/f1000research.160396.1

**Published:** 2025-01-15

**Authors:** Oam To-aj, Apithai Bumrungpanictarworn, Felix Liauw

**Affiliations:** 1 Faculty of Social Sciences and Humanities, Mahidol University, Nakhon Pathom, Thailand; 2 Department of Health Science, Macquarie University, Sydney, Australia

**Keywords:** Table Tennis, Competitive training, Recreation sport, Quality of life, Older adults

## Abstract

**Background:**

Owing to its combination of speed, tactics, and accuracy, table tennis is becoming a sport exercise that is suitable for older adults. Casual play is fun and relaxing, and more serious table tennis might further develop skills through focused practice.

**Methods:**

A randomized controlled trial (RCT) aimed to determine how the practice of competition compared to leisure playing affects the Quality of life (QoL) of people aged 40-70. Selecting subjects at random, one group of subjects undertook a structured competitive regimen, whereas the other group was given time to play recreationally without any structured competition. QoL was measured before and after the intervention using the WHOQOL-BREF, which measures physical, psychological, social, and environmental domains. Data were analyzed using paired and independent t-test.

**Results:**

The group that received structured competition reported a marked improvement in their QoL indicators, especially in physical health (p < 0.001), psychological health (p < 0.001), and social relationships (p = 0.001), when compared to the control group, which showed few improvements. No significant differences were observed in the environmental domains in either group.

**Conclusions:**

Regular competitive table tennis practice improves physical condition, emotions, and social relationships, which leads to a higher QoL in older adults. Thus, these results emphasize the utility of competitive sports in the maintenance of active and healthy aging.

**Registration:**

Thai Clinical Trial Registry (TCTR) ID TCTR20250105001, registered on December 28, 2024

## Introduction

Aging is a natural process that is frequently linked with challenges in the physical, cognitive, and social domains, resulting in a decline in the overall quality of life (QoL) ( Huntula et al., 2022; Saxon et al., 2021). With the rapidly increasing number of elderly individuals worldwide, the need to maintain a healthy life has become a serious issue ( Lunenfeld & Stratton, 2013). Physical activity is widely recognized as an effective intervention to mitigate the effects of aging, particularly by addressing physical decline and enhancing overall well-being ( Taylor et al., 2004). Research has shown that exercise has a stronger ability to ameliorate biological alterations associated with aging, such as excessive inflammation, and arguably working towards restoration of overall health and improvement of aging ( Huntula et al., 2022). It is important to create an environment that can improve these aspects in older adults to enhance their overall life and sense of autonomy ( Nordenfelt, 2009).

Moderate to vigorous physical activity and vigorous exercise have several advantages, especially in enhancing health and reducing the impact of aging ( Taylor et al., 2004). Physical activity is beneficial for enhancing physical health, psychological well-being, and social participation ( McPhee et al., 2016; Taylor et al., 2004). Nevertheless, research on the effect of different types of exercise on the well-being of older adults is still a very relevant field of study ( Sun et al., 2013). In particular, recreational sports are becoming interesting through the promotion of movement and exercise, thinking, and other socializing aspects ( Berg et al., 2015).

Table tennis is a sport that requires quickness in deciding, coordination, and agility, is an effective exercise among the older adult since it is low impact, can be modified if possible for both the seriousness and fun of the game ( He et al., 2022). While recreational table tennis is often played for fun, competition and its related training requires a more serious approach which utilizes designed practices that can be beneficial too ( Naderi et al. 2018; Shields & Bredemeier, 2009). In addition, sport for recreation can simply engage in various physical activities, in contrast with sport for competition that requires setting aims, exercising self control, and putting more efforts, these goals enable building stronger character components such as endurance, concentration, kind of success, and may improve overall QoL ( Locke & Latham, 1985; McCormick et al., 2019).

As for physical activity interventions that have already been researched, they mainly involved generic exercise forms such as walking, yoga, and strength training, as they focus on engaging the core to enhance mobility and independence of older adults so that they can carry out their daily tasks more easily ( Abdin et al., 2019; Foster & Armstrong, 2018; Saadprai et al., 2021). Although these activities have shown positive results, they might not provide the same multifaceted engagement as competitive sports do ( Foster & Armstrong, 2018). In addition, the differences in the benefits enjoyed by recreation and competitive sports participants remain uninvestigated. Whereas recreational play might focus more on pleasure and relaxation, competitive training provides a particular context that gives individuals the desire to reach specific results ( Dixon et al., 2020). This framework might further enhance QoL by instilling a sense of direction, order, and achievement.

This study attempts to fill this gap by examining the impact of engaging in competitive table tennis training along with recreation on the QoL of older adults. In this randomized controlled trial (RCT), participants in the experimental group underwent a structured training program led by expert coaches, focusing on competitive skills and strategies. On the other hand, the control group practiced unorganized leisure table tennis activities. In this instance, we will compare the outcomes of the QoL measurements of the two groups and attempt to determine whether the competitive training approach is more advantageous than the recreational approach for improving QoL.

### Objectives

1. To determine the difference in the QoL of the experimental group after they have been involved in a formulated competitive table tennis training schedule.
2. To measure and analyse changes on the post-intervention QoL between subjects in the experimental group and the subjects in the control group.
3. To determine whether competitive training provides greater improvements in QoL than recreational play.

## Methods

Registration: Thai Clinical Trial Registry (TCTR) ID TCTR20250105001, registered on December 28, 2024, < https://www.thaiclinicaltrials.org/show/TCTR20250105001>.

### Study design

This study was a randomized controlled trial (RCT) with two groups: a structured competitive table tennis training group and a recreational play group, with the aim of assessing the impact of competitive training on the quality of life of seniors aged 40 and above. QoL assessments should be conducted at the beginning of the intervention and after 30 days of intervention.

### Participants and setting

The study was conducted at the TTAT Academy in Bangkok, Thailand, and data were collected from the participants in the 1st of August to 30th of August 2024. The participants were recruited from local community centers and table tennis clubs in Bangkok and were aged between 40 and 70 years. The trial was randomized and included 31 participants in total, of which 16 were placed in the experimental group and 15 were placed in the control group. From the recruited 31 participants, 18 were male and 13 were female, which included 10 males and 6 females in the experimental group and 8 males and 7 females in the control group.

In this study, the age group of 40–70 years was selected because of competition protocols that define any person over 40 years of age as an older adult. Such classification and categorization have been adopted in international competitions governed by the International Table Tennis Federation ( International Table Tennis Federation, n.d.); hence, this age group is immensely useful in determining the impact of competitive table tennis training on QoL. 

This study was approved by the university board and university ethics committee, and all participants provided informed consent prior to the study. Mostly physical ability and knowledge of table tennis was a requirement for participation. However, participants were excluded from the study if they had prior professional experience with table tennis, severe cognitive or physical disabilities, or any medical conditions that limited their recreational activities.

### Randomization and allocation

Participants were assigned to either the experimental or control group using simple randomization. During this process, participants were assigned to a group based on a random draw. Thus, each participant had an equal probability of being allocated to either group. Informed consent was obtained first and baseline assessments were performed. Only then were participants told which intervention group they belonged to, in order to retain concealment of allocation. This method minimized selection bias and hindered the randomization process.

### Experimental group

For a period of 30 days, the participants in the experimental group were subjected to competitive table tennis training through allocated sessions on a daily basis. The time frame was fixed from 10 am to 12 pm and from 3 pm to 5 pm as two training sessions for each day. Each session was structured using a fifteen minute warm up timeline that contained aerobic exercises and dynamic stretches to prepare the body for rigorous training and reduce the risk of injuries. This was followed by a thirty minute instructional course that focused on crucial table tennis skills to be developed, such as forehand and backhand strokes, spins, and footwork.

After that, a fifteen minute hydration and rest break was incorporated into the routine and then moved on to a muscle-building course in order to strengthen their strategies such as offense, defense, positioning, and general gameplay for 30 min. Then a fifteen minute physical conditioning program was added to strengthen their speed, endurance, and agility. The last final fifteen minutes of this training session combined muscle relaxation with static stretching and also allowed the participants to reflect and reinforce on the main points of the day’s training. To conclude the program, the final two days simulated professional competitive matches with teams and singles to give a taste of real tournaments to the participants.

### Control group

The control group participants performed recreational table tennis with the same duration and frequency of hours played in the experimental group. No feedback or structured coaching was offered to the players. These sessions focused on social interaction and enjoyment rather than a professional setting.

### Procedure

1. Recruitment and Consent: Sports clubs and local community centers were used as recruiting grounds for participants with active announcements. Before participants were enrolled into the study, consent was obtained from all enrolled participants.
2. Baseline Assessment: The WHOQOL-BREF questionnaire was given to the participants as an assessment measuring QoL before the interventions began to take place.
3. Intervention Implementation: There were descriptions and control protocols stating exactly what the participants were required to do and the interventions set in place.
4. Post-Intervention Assessment: The participants completed the intervention period of 30 days after which the WHOQOL-BREF questionnaire is filled out to assess

### Outcome measures

The primary outcome was the change in QoL assessed through the WHOQOL-BREF questionnaire, which was developed by the World Health Organization (WHO). This standardized tool for evaluating QOL encompasses four aspects: physical, psychological, social, and environmental. Each domain is scored on a scale of 1 to 5, with higher scores reflecting better QoL ( World Health Organization, n.d.). The total QoL score is the sum cost of the four domains, resulting in a total score of 130–26 and described as poor QoL, 26–60; moderate QoL, 61–95; and good QoL, 96–130.

### Physical health

This domain examined mobility, daily activity, sleep, and levels of discomfort. The maximum score was 35, while less than 16 was classified as poor, 17–26 as moderate, and above 27 as good.

### Psychological health

This domain assesses the better self, including self-esteem, concentration, positive and negative affect, and emotional health. The maximum score was 30, while less than 14 was classified as poor, 15–22 as moderate, and 23 and above as good.

### Social relationships

This domain assessed family relationships, social interactions, and satisfaction with social interactions. The maximum score was 15, while less than 7 was classified as poor, 8–11 as moderate, and above 12 as good.

### Environmental factors

Previous work has defined QoL as perceived safety and security, availability of resources, economic conditions, and environmental conditions. The maximum score was 40, where a score lower than 18 was considered poor, 19–29 is moderate, and above 30 was good.

The reliability already shown throughout the years renders the WHOQOL-BREF credible for the assessment of QoL across diverse populations. This accounts for its selection in this study.

### Data collection and analysis

The data that were collected used two intervals: the first one at baseline, which was done before the intervention, and the second one after the intervention day, which was day 30. It would then be uploaded to an electronic database and then analyzed by the help of the IBM SPSS Statistics Vol 22 software. Descriptive statistics were used to summarize baseline characteristics, with categorical variables recorded as frequency and percentage and continuous variables as mean and standard deviation.

The paired t-test determined within-group changes occurring in QoL scores of the samples in the two periods of pre-intervention and post-intervention in the experimental and control group participants across four domains: physical health, psychological health, social relationships, and environmental factors. Independent t-tests were also necessary to compare the mean post-interventional QoL scores between the experimental and control groups.

Chi-squared test analysis was performed to assess the QoL – poor, moderate, or good levels of each group pre-intervention and post-intervention in both groups. The means and their 95% confidence intervals (CIs) were also determined regarding the differences between pre-intervention and post-intervention to assess whether there were any statistically significant differences. The standard for a statistically significant difference was set at p < 0.05. I would like to know how the study was approved by the appropriate bodies, as obtaining such approvals can sometimes be challenging.

Ethical approval was obtained The Committee for Research Ethics (Social Sciences), Faculty of Social Sciences and Humanities, Mahidol University, under approval number 2023/205.3011, date of approval on November 30, 2023. The Committee for Research Ethics (Social Sciences) strictly adheres to international guidelines for human subject protection, including the Declaration of Helsinki, the Belmont Report, and the CIOMS guidelines. Written informed consent was obtained from all participants who were provided with detailed information about the study’s purpose and procedures. They were assured that their data would be strictly used for research purposes and publications. Participants retained the right to withdraw from the study at any time, without any consequences. However, all participants completed the study without any withdrawal.

### Clinical trial registration

This study was registered with Thai Clinical Trials Registry (TCTR) on December 28, 2024 (TCTR20250105001). Retrospective registration occurred because the research team prioritized completing the study within the project timeline and there was a shift in the study location, which required some modifications. As a result, registration cannot be completed in time. However, this has now been completed to ensure transparency and compliance with international standards.

## Result

### Participants’ demographic and baseline characteristics

**Figure 1.**
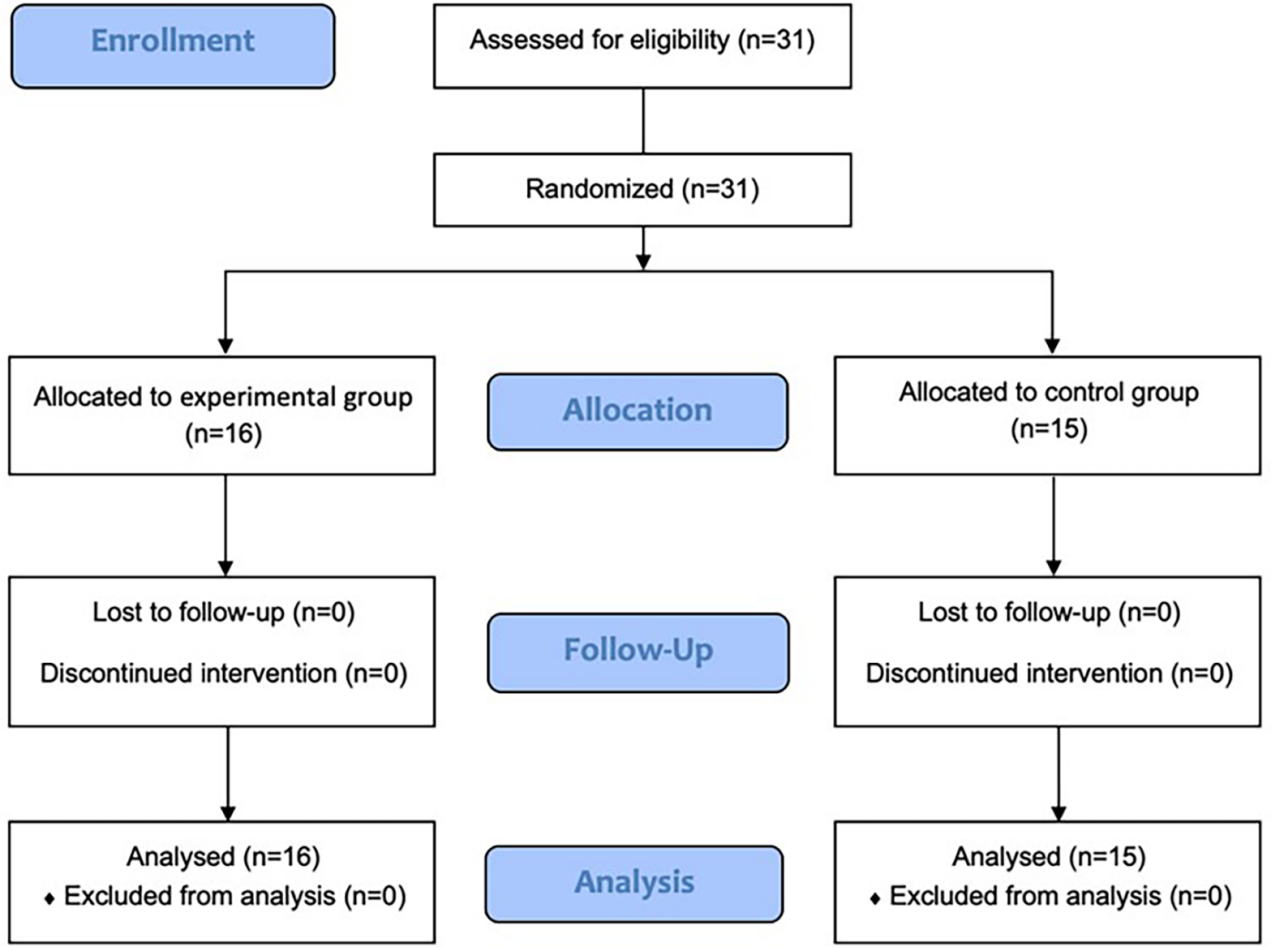
Study flow diagram.

The study comprised 31 participants, with 16 and of experimental and control participants, respectively ( Figure 1). The age of the participants ranged with mean being 56.65 and standard deviation being 7.97 however, specifically the experimental group was significantly younger in comparison with the mean age of being 53.31 (SD = 7.67) while the control group possessed an average of mean age of 60.20 (SD = 6.85) with p value of 0.024. The study included more male participants (58.06%), with the majority being ten males (62.5%) in the experimental group and eight males (53.3%) in the control group. There were no significant differences other than the mean age of the participants in terms of baseline characteristics between the experimental and control groups. There was an age gap of 6.9 years between the two groups, where the control participants averaged older ( Table 1).

**Table 1.** Participants’ demographic.

Characteristic	Total (n = 31)	Experimental Group (n = 16)	Control Group (n = 15)	p-value
**Sex** (n (%))				
Male	18 (58.1%)	10 (62.5%)	8 (53.3%)	0.621
Female	13 (41.9%)	6 (37.5%)	7 (46.7%)	
**Age** (Mean (SD))	56.65 (7.97)	53.31 (7.67)	60.20 (6.85)	0.024*

The QoL baseline scores of the groups differed from those of the experimental group, outperforming the control group with an overall QoL of 95.38 (SD = 6.75) in comparison with 88.40 (SD = 5.75), p = 0.045). Such significant differences were noted in the physical health and social relationship domains but otherwise followed suit with no major differences witnessed (see Table 2).

**Table 2.** Baseline characteristics.

Baseline QoL (Mean (SD))	Total (n = 31)	Experimental Group (n = 16)	Control Group (n = 15)	p-value
Overall QoL	91.89 (6.25)	95.38 (6.75)	88.40 (5.75)	0.045*
Physical Health	27.63 (3.01)	27.63 (3.01)	25.07 (2.46)	0.045*
Psychological Health	23.94 (2.08)	23.94 (2.08)	23.27 (2.58)	0.501
Social Relationships	11.81 (1.05)	11.81 (1.05)	10.13 (0.92)	0.028*
Environmental Factors	32.00 (3.61)	32.00 (3.61)	29.93 (3.41)	0.067

### Primary outcomes

Statistical analysis revealed that the experimental group showed a pronounced enhancement in the overall concentration. The rating was improved from 95.38 (SD = 6.75) at the start to 101.13 (SD = 7.99) at the end of the procedure with p value being lower than 0.001. In contrast, the control group did not show any improvement, with averages improving from 88.40 (SD = 5.75) to 89.53 (SD = 5.51; Table 3).

**Table 3.** Changes in QoL scores after the intervention.

Domain	Pre-Test (Mean (SD))	Pre-Test (Mean (SD))	Post-Test (Mean (SD))	Post-Test (Mean (SD))	p-value (Pre vs. Post)
	Experimental	Control	Experimental	Control	
Overall QoL	95.38 (6.75)	88.40 (5.75)	101.13 (7.99)	89.53 (5.51)	< 0.001***
Physical Health	27.63 (3.01)	25.07 (2.46)	30.25 (2.70)	25.73 (2.05)	< 0.001***
Psychological Health	23.94 (2.08)	23.27 (2.58)	25.63 (2.60)	23.60 (2.26)	< 0.001***
Social Relationships	11.81 (1.05)	10.13 (0.92)	13.25 (1.00)	10.27 (0.96)	0.001**
Environmental Factors	32.00 (3.61)	29.93 (3.41)	32.00 (3.61)	29.93 (3.41)	> 0.05

### Physical health

Statistical data indicated that active members in the experimental group showed considerable improvement in the overall physical health rating going from 27.63 (SD = 3.01) to 30.25 (SD = 2.70), which had a p-value of < 0.001. Similarly, the control group also did not report a drastic change, with results improving from 25.07 (SD = 2.46) and new ratings of 25.73 (SD = 2.05) pacing at p = 0.134.

### Psychological health

In contrast with the control group that experienced a moderate non-significant upturn from an average of 23.27 (SD = 2.58) to 23.60 (SD = 2.26), p value being 0.096, the experimental group noted a visible shift on the charts with an average that improved from 23.94 (SD = 2.08) to 25.63 (SD = 2.60) with a p-value of lower than 0.001.

### Social relationships

The results showed that there was a notable improvement for the experimental group, raising their scores from 11.81 (SD = 1.05) to 13.25 (SD = 1.00) with a p value of 0.001. The control group only saw a minimal improvement from 10.13 (SD = 0.92) to 10.27 (SD = 0.96) for a p-value of 0.164.

### Environmental factors

None of the groups showed any major differences in this regard. The experimental group remained constant with a score of 32.00 (SD = 3.61) and the control group at 29.93 (SD = 3.41), with a p value of 0.05.

### Between-group comparisons

As seen in Table 3, the report shows that there were major differences in the experimental and control groups’ QoL and in three of the specific areas that were assessed. The mean scores for the permanent experimenters were comparatively higher than those for the control group (101.13 vs. 89.53, p < 0.001). The results in the above item regarding Physical Health showed that the experimental group scored higher than the control group (30.25 vs 25.73, p < 0.001). As in the Experimental Group, where the scores were also higher in Psychological Health (25.63 vs 23.60, p = 0.029) and Social Relationships (13.25 vs 10.27, p = 0.001). However, the two groups did not show any major differences in Environmental Factors (p > 0.05).

### Proportion of QoL levels

In the experimental group, the proportion of participants with a ‘good’ QoL (96–130) increased from 37.50% at baseline to 68.75% post-intervention. On the contrary, the control group did not have any considerable differences with only 6.67% being able to attain ‘good’ QoL at both periods. Most importantly, the experimental group was able to achieve ‘good’ QoL with regards to the social relationships domain, with 100% able to achieve the results in contrast to 13.33% of the control group post-intervention Table 4.

**Table 4.** Proportion of QoL Levels (Pre-Test and Post-Test).

QoL Level	Pre-Test % (Experimental)	Pre-Test % (Control)	Post-Test % (Experimental)	Post-Test % (Control)
Poor (26–60)	0	0	0	0
Moderate (61–95)	62.5	93.33	31.25	93.33
Good (96–130)	37.5	6.67	68.75	6.67

## Discussion

This study aimed to investigate the effects of a structured table tennis intervention on QoL. After careful observation of the intervention, it was established that the psychological and physical health of the older adults in the experimental group significantly improved. No significant changes were observed in the control group. According to data from existing studies, sports participation and its life quality benefits for older adults have persisted ( Acree et al., 2006). In contrast to ordinary exercises, sports competitions, such as table tennis, add a different dimension to mental and social activities, such as well-structured training, rigorous thought processes, and precise interactions ( Bar-Eli et al., 2011).

Most importantly, the considerable improvement in physical health scores for the experimental group highlights the role of table tennis in meeting physical requirements and providing cardiovascular benefits. According to prior research, competing in organized sports helps enhance physical health parameters by ameliorating cardiovascular function and the development of muscle structure ( Schmidt et al., 2015). These benefits are in agreement with previous observations, indicating the scope of table tennis as a tool for enhancing physical health in general ( Mahesh et al., 2013; Naderi et al., 2018). Additionally, the competitive aspect of the training protocol may have provided additional benefits, since all the subjects were raised with the goal of improving their skill and physical performance, which made the process more interesting.

Such psychological effects observed in the present study are consistent with the results of other studies, which suggest that participating in competitive sports enhances emotional well-being, minimizes depression, and increases self-confidence ( Andersen et al., 2019). Such phenomena may be due to the fact that such training is rather structured with clear objectives, thereby giving the trainees a sense of accomplishment. Furthermore, the improvements observed in the experimental group align with previous studies that demonstrated the role of structured physical activities in enhancing psychological resilience and reducing biological stress markers ( Mitarnun et al., 2022). These findings further emphasize the advantages of competitive sports in promoting mental health, as evidenced by the changes in the Psychological Health domain in the experimental group, which supports the aforementioned claims.

The results of this study support the claims of Sánchez-Santos et al. (2024) that there is an enhancement of social networks for older adults engaging in sporting events. Similar to the results of this study, the role of competitive sports in creating social interaction, particularly with other competitive sports participants. The findings from this study suggest that organized training provided the participants with opportunities to socialize, which in turn enhanced social interactions, thereby improving their QoL.

It is worth noting that the established changes in the environmental factors in both groups were not statistically significant. This finding may represent the overshadowing of short-term intervention approaches on wider environmental aspects, such as resource availability or security perceptions. Further studies are needed to consider ways of combining physical activity strategies with intervention strategies to address these issues.

It needs to be clarified whether there is a distinction between competitive training and leisure physical activity. Protocols were set for training, which would aid the subjects in training at the level of a competitive table tennis player. Additionally, it stressed the need for increased concentration, self-control, and resolution to practice at an intense level of competition, which is very different from recreation table tennis. These variations may explain the remarkable alterations that occurred in the physical and psychological domains of the experimental group.

## Conclusion

The present study highlighted that the practice of competitive table tennis has a meaningful effect on the health-related QoL of older adults, especially in the physical, psychological, and social dimensions. Such systematic and goal-setting training as a competitive training has additional benefits that are not gained in the case of recreational play, lending credence to its efficacy as a means of enhancing health and well-being among older adults. Moreover, this study demonstrated the effectiveness of integrating competitive sporting activities into health programs and additional interventions targeted at older people.

### Strengths and limitations

One of the most remarkable advantages of this study is that it has a randomized controlled design that minimizes bias, thereby adding credibility to the findings. In addition, the adoption of the WHOQOL-BREF, an instrument that has been validated for QoL assessment, ensures the reliability and comparability of findings across studies. This study has certain limitations. First, the sample of respondents was rather small, which might restrict the generalizability of the findings. Second, the relatively shorter period of intervention (30 days) can be said to affect the range of effects that the game of table tennis has on QoL. A longer follow-up period should be considered in future studies to evaluate the strength of the observed changes.

### Implications and future directions

Therefore, this analysis provides scope for utilizing table tennis as a tool to improve QoL among older adults in an enjoyable manner. It is recommended that similar approaches are adopted in the community health domain as they would be cost-effective and convenient for the promotion of health in this demographic. Future studies should incorporate individuals with chronic illnesses or disabilities into the interventions to investigate the impact of table tennis across larger populations. Furthermore, there is a lack of understanding regarding the long-term effects of such interventions on health and QoL in older adults; therefore, these requirements need to be fulfilled.

## Ethics and consent

This research received ethical approval from the Office of the Committee for Research Ethics (Social Sciences), Faculty of Social Sciences and Humanities, Mahidol University, under approval number 2023/205.3011. The Committee for Research Ethics (Social Sciences) strictly adheres to international guidelines for human subject protection, including the Declaration of Helsinki, the Belmont Report, and the CIOMS guidelines. This study was registered in the Thai Clinical Trials Registry (TCTR) on December 28, 2024 (TCTR20250105001). Retrospective registration occurred because the research team prioritized completing the study within the project timeline and there was a shift in the study location, which required some modifications. As a result, registration cannot be completed in time. However, this has now been completed to ensure transparency and compliance with international standards. Written informed consent was obtained from all participants who were provided with detailed information about the study’s purpose and procedures. They were assured that their data would be strictly used for research purposes and publications. Participants retained the right to withdraw from the study at any time, without any consequences. However, all participants completed the study without any withdrawal.

## Data Availability

Figshare: Quality of Life Score for Experimental and Control Group (Pre and Post), https://doi.org/10.6084/m9.figshare.28079462.v2 ( To-aj et al., 2024). The project contains the following underlying data:
• Experimental and Control Group QoL Score Data are available under the terms of the Creative Commons Attribution 4.0 International license (CC-BY 4.0). Figshare: CONSORT Checklist and Study Flow Diagram - Comparing Competitive and Recreational Table Tennis Training: Impacts on Quality of life in Older Adults - A Randomized Controlled Trial, DOI: https://doi.org/10.6084/m9.figshare.28152722.v2 ( To-aj et al., 2025). The project has the following reporting guidelines:
• CONSORT Checklist Competitive Table Tennis • CONSORT-Flow chart Data are available under the terms of the Creative Commons Attribution 4.0 International license (CC-BY 4.0).

## References

[ref1] Abdin S Lavallee JF Faulkner J : A systematic review of the effectiveness of physical activity interventions in adults with breast cancer by physical activity type and mode of participation. *Psycho-Oncology.* 2019;28(7):1381–1393. 10.1002/pon.5101 31041830

[ref2] Acree LS Longfors J Fjeldstad AS : Physical activity is related to quality of life in older adults. *Health Qual. Life Outcomes.* 2006;4:1–6. 10.1186/1477-7525-4-37 16813655 PMC1524938

[ref3] Andersen MH Ottesen L Thing LF : The social and psychological health outcomes of team sport participation in adults: An integrative review of research. *Scand. J. Public Health.* 2019;47(8):832–850. 10.1177/1403494818791405 30113260

[ref4] Bar-Eli M Plessner H Raab M : *Judgment, decision-making and success in sport.* John Wiley & Sons;2011; vol.1. 10.1002/9781119977032

[ref5] Berg BK Warner S Das BM : What about sport? A public health perspective on leisure-time physical activity. *Sport Management Review.* 2015;18(1):20–31. 10.1016/j.smr.2014.09.005

[ref6] Dixon JG Jones MV Turner MJ : The benefits of a challenge approach on match day: Investigating cardiovascular reactivity in professional academy soccer players. *Eur. J. Sport Sci.* 2020;20(3):375–385. 10.1080/17461391.2019.1629179 31167615

[ref7] Foster C Armstrong ME : What types of physical activities are effective in developing muscle and bone strength and balance? *J. Frailty Sarcopenia Falls.* 2018;03(2):58–65. 10.22540/JFSF-03-058 32300694 PMC7155324

[ref8] He Y Shao S Fekete G : Lower Limb Muscle Forces in Table Tennis Footwork during Topspin Forehand Stroke Based on the OpenSim Musculoskeletal Model: A Pilot Study. *Mol. Cell. Biomech.* 2022;19(4):221–235. 10.32604/mcb.2022.027285

[ref9] Huntula S Lalert L Punsawad C : The Effects of Exercise on Aging-Induced Exaggerated Cytokine Responses: An Interdisciplinary Discussion. *Scientifica.* 2022;2022(1):3619311–3619362. 10.1155/2022/3619362 35106183 PMC8801319

[ref10] International Table Tennis Federation: ITTF World Masters Championships Rome 2024. n.d. Reference Source

[ref11] Locke EA Latham GP : The application of goal setting to sports. *J. Sport Exerc. Psychol.* 1985;7(3):205–222. 10.1123/jsp.7.3.205

[ref12] Lunenfeld B Stratton P : The clinical consequences of an ageing world and preventive strategies. *Best Pract. Res. Clin. Obstet. Gynaecol.* 2013;27(5):643–659. 10.1016/j.bpobgyn.2013.02.005 23541823 PMC3776003

[ref13] Mahesh B Kalpesh V Priti B : A comparative study of visual reaction time in table tennis players and healthy controls. *Indian J. Physiol. Pharmacol.* 2013;57(4):439–442. 24968584

[ref14] McCormick A Meijen C Anstiss PA : Self-regulation in endurance sports: Theory, research, and practice. *Int. Rev. Sport Exerc. Psychol.* 2019;12(1):235–264. 10.1080/1750984X.2018.1469161

[ref15] McPhee JS French DP Jackson D : Physical activity in older age: perspectives for healthy ageing and frailty. *Biogerontology.* 2016;17:567–580. 10.1007/s10522-016-9641-0 26936444 PMC4889622

[ref16] Mitarnun W Mitranun W Mitarnun W : Home-based walking meditation decreases disease severity in Parkinson’s disease: a randomized controlled trial. *J. Integr. Complement. Med.* 2022;28(3):227–233. 10.1089/jicm.2021.0292 35294297

[ref17] Naderi A Degens H Rezvani MH : A retrospective comparison of physical health in regular recreational table tennis participants and sedentary elderly men. *J. Musculoskelet. Neuronal Interact.* 2018;18(2):200–207. Reference Source 29855442 PMC6016501

[ref18] Nordenfelt L : *Dignity in care for older people.* Blackwell Publishing Ltd.;2009.

[ref19] Saadprai S Silapabanleng S Phengjam M : Design and development of a core strengthening exercercise and rehabilitation machine for elderly. *Suranaree J. Sci. Technol.* 2021;28(3):1–7. Reference Source

[ref20] Sánchez-Santos JM Rungo P Lera-López F : Building social capital through sport engagement: Evidence for adults aged 50 years and older. *Ageing Soc.* 2024;44(2):403–428. 10.1017/S0144686X22000046

[ref21] Saxon SV Etten MJ Perkins EA : *Physical change and aging: A guide for helping professions.* Springer Publishing Company;2021.

[ref22] Schmidt JF Andersen TR Andersen LJ : Cardiovascular function is better in veteran football players than age-matched untrained elderly healthy men. *Scand. J. Med. Sci. Sports.* 2015;25(1):61–69. 10.1111/sms.12153 24303918

[ref23] Shields DL Bredemeier BL : *True competition.* Human Kinetics;2009.

[ref24] Sun F Norman IJ While AE : Physical activity in older people: a systematic review. *BMC Public Health.* 2013;13:1–17. 10.1186/1471-2458-13-449 Reference Source 23648225 PMC3651278

[ref25] Taylor AH Cable NT Faulkner G : Physical activity and older adults: a review of health benefits and the effectiveness of interventions. *J. Sports Sci.* 2004;22(8):703–725. 10.1080/02640410410001712421 15370483

[ref26] To-aj O Bumrungpanictarworn A Liauw F : Quality of Life Score for Experimental and Control Group (Pre and Post).Dataset. *figshare.* 2024. 10.6084/m9.figshare.28079462.v2

[ref27] To-aj O Bumrungpanictarworn A Liauw F : CONSORT Checklist and Study Flow Diagram - Comparing Competitive and Recreational Table Tennis Training: Impacts on Quality of life in Older Adults - A Randomized Controlled Trial.Dataset. *figshare.* 2025. 10.6084/m9.figshare.28152722.v2 PMC12205305 40584892

[ref28] World Health Organization: WHOQOL: Measuring Quality of Life. n.d. Reference Source

