# Supplementary material for: Comparing Competitive and Recreational Table Tennis Training: Impacts on Quality of life in Older Adults - A Randomized Controlled Trial
Source: F1000Res. 2025 Jun 25;14:89. Originally published 2025 Jan 15. [Version 2] doi: 10.12688/f1000research.160396.2 (PMC12205305; doi:10.12688/f1000research.160396.2)
Supplement: Peer review history

# Reviewer report: Reviewer response for version 2

**DOI:** 10.5256/f1000research.183926.r394765

**Published:** 2025-06-28

**Authors:** Sairag Saadprai

**Affiliations:** 1 Department of Sports Science and Sports Development, Faculty of Allied Health Sciences, Thammasat university, Bangkok, Thailand

I have no further comments to make.

Is the work clearly and accurately presented and does it cite the current literature?

Partly

If applicable, is the statistical analysis and its interpretation appropriate?

Yes

Are all the source data underlying the results available to ensure full reproducibility?

Yes

Is the study design appropriate and is the work technically sound?

Yes

Are the conclusions drawn adequately supported by the results?

Partly

Are sufficient details of methods and analysis provided to allow replication by others?

Partly

Reviewer Expertise:

My area of research is Sport Science.

I confirm that I have read this submission and believe that I have an appropriate level of expertise to confirm that it is of an acceptable scientific standard.

# Reviewer report: Reviewer response for version 2

**DOI:** 10.5256/f1000research.183926.r394764

**Published:** 2025-06-27

**Authors:** Hadeel Halaweh

**Affiliations:** 1 Al-Quds University, Jerusalem, Palestinian Territory

Thank you for considering my comments.

 My decision is approved

Is the work clearly and accurately presented and does it cite the current literature?

Partly

If applicable, is the statistical analysis and its interpretation appropriate?

Partly

Are all the source data underlying the results available to ensure full reproducibility?

Partly

Is the study design appropriate and is the work technically sound?

Partly

Are the conclusions drawn adequately supported by the results?

Partly

Are sufficient details of methods and analysis provided to allow replication by others?

Partly

Reviewer Expertise:

Quality of life, gerontology, physical activity.

I confirm that I have read this submission and believe that I have an appropriate level of expertise to confirm that it is of an acceptable scientific standard.

# Reviewer report: Reviewer response for version 1

**DOI:** 10.5256/f1000research.176288.r377811

**Published:** 2025-06-17

**Authors:** Sairag Saadprai

**Affiliations:** 1 Department of Sports Science and Sports Development, Faculty of Allied Health Sciences, Thammasat university, Bangkok, Thailand

This manuscript presents a clearly written and timely investigation into the effects of competitive versus recreational table tennis training on quality of life in older adults, using an appropriate randomized controlled trial design. It addressed a relevant problem, applied suitable methodology, and the results were supported by sound statistical analysis and presented in a clear manner.

 There are a few areas that require minor but necessary revisions before the manuscript can be considered ready for indexing. These include updating selected references, optionally reporting effect size metrics, clarifying the tone in the conclusions, and removing a sentence that appears to have been included in error.

 Overall, this is a valuable and well-executed study that contributes meaningfully to the field of physical activity and aging. I recommend the article for approval with reservations.

 I would like to offer the following suggestions to enhance clarity and completeness as follow:

 1. Literature Update

 Some of the cited references are dated. The authors are encouraged to include more recent literature (preferably from the past five years) to further support the study’s rationale especially regarding the mental and social health benefits of structured physical activity in older adults.

 2. Effect Size Reporting

 While the statistical analyses are appropriate and well-applied, the manuscript would benefit from reporting effect size measures to help readers better understand the magnitude of differences between groups.

 3. Language and Tone in the Conclusions

 The authors may consider slightly softening the tone of the conclusions and clearly acknowledging the study’s limitations (e.g., age imbalance between groups and short intervention duration) to maintain a balanced and cautious interpretation of the results.

 4. Unintended Sentence in the Methods Section

 The following sentence appears to have been included by mistake, as it uses first-person voice, which is generally discouraged in academic writing. It should be removed for consistency with the overall tone of the manuscript: “I would like to know how the study was approved by the appropriate bodies, as obtaining such approvals can sometimes be challenging.”

Is the work clearly and accurately presented and does it cite the current literature?

Partly

If applicable, is the statistical analysis and its interpretation appropriate?

Yes

Are all the source data underlying the results available to ensure full reproducibility?

Yes

Is the study design appropriate and is the work technically sound?

Yes

Are the conclusions drawn adequately supported by the results?

Partly

Are sufficient details of methods and analysis provided to allow replication by others?

Partly

Reviewer Expertise:

My area of research is Sport Science.

I confirm that I have read this submission and believe that I have an appropriate level of expertise to confirm that it is of an acceptable scientific standard.

# Reviewer report: Reviewer response for version 1

**DOI:** 10.5256/f1000research.176288.r363782

**Published:** 2025-02-17

**Authors:** Hadeel Halaweh

**Affiliations:** 1 Al-Quds University, Jerusalem, Palestinian Territory

Thank you for the opportunity to review this manuscript entitled “ Comparing Competitive and Recreational Table Tennis Training: Impacts on Quality of life in Older Adults - A Randomized Controlled Trial”.

 Introduction:

- Generally well-written section.
- I noticed an overlap among the three listed objectives and suggest revising them into a single, consolidated objective.

Methods:

- Please clarify who conducted the randomization.
- Which language was used for the WHOQOL-BREF questionnaire? Was it a validated version? If so, please provide a reference.
- The training sessions were scheduled from 10 AM to 12 PM and 3 PM to 5 PM each day. Please clarify whether each participant attended a total of four hours of training daily for 30 days. Additionally, how did you address the risk of exhaustion and fatigue, considering the participants' age range of 40–70 years?
- In the data collection section, There is a sentence that has to be deleted “I would like to know how the study was approved by the appropriate bodies, as obtaining such approvals can sometimes be challenging”. Please verify.
- I would prefer it if you have a separate title for ethical considerations.

Results:

- A major concern in this study is the significant age difference between the experimental and control groups, with the experimental group being younger (p = 0.024). This discrepancy may compromise the internal validity of the results, as age itself can be an independent factor influencing QoL, with younger individuals potentially exhibiting better physical performance.
- As shown in Table 2, there were significant differences between the groups in overall QoL, physical health, and social relationships. This raises concerns about the internal validity of the study, particularly regarding the impact of the independent variable (structured training) on QoL domains.
- Please calculate the effect size.

Is the work clearly and accurately presented and does it cite the current literature?

Partly

If applicable, is the statistical analysis and its interpretation appropriate?

Partly

Are all the source data underlying the results available to ensure full reproducibility?

Partly

Is the study design appropriate and is the work technically sound?

Partly

Are the conclusions drawn adequately supported by the results?

Partly

Are sufficient details of methods and analysis provided to allow replication by others?

Partly

Reviewer Expertise:

Quality of life, gerontology, physical activity.

I confirm that I have read this submission and believe that I have an appropriate level of expertise to confirm that it is of an acceptable scientific standard, however I have significant reservations, as outlined above.
